# Prediction of anticancer drug sensitivity using an interpretable model guided by deep learning

**DOI:** 10.1186/s12859-024-05669-x

**Published:** 2024-05-09

**Authors:** Weixiong Pang, Ming Chen, Yufang Qin

**Affiliations:** 1 College of Information Technology, Shanghai Ocean University, Hucheng Ring Road, Shanghai, China; 2 Key Laboratory of Fisheries Information Ministry of Agriculture, Shanghai, China

**Keywords:** Drug sensitivity, Deep neural networks, Interpretability model, Biological pathway

## Abstract

**Background:**

The prediction of drug sensitivity plays a crucial role in improving the therapeutic effect of drugs. However, testing the effectiveness of drugs is challenging due to the complex mechanism of drug reactions and the lack of interpretability in most machine learning and deep learning methods. Therefore, it is imperative to establish an interpretable model that receives various cell line and drug feature data to learn drug response mechanisms and achieve stable predictions between available datasets.

**Results:**

This study proposes a new and interpretable deep learning model, DrugGene, which integrates gene expression, gene mutation, gene copy number variation of cancer cells, and chemical characteristics of anticancer drugs to predict their sensitivity. This model comprises two different branches of neural networks, where the first involves a hierarchical structure of biological subsystems that uses the biological processes of human cells to form a visual neural network (VNN) and an interpretable deep neural network for human cancer cells. DrugGene receives genotype input from the cell line and detects changes in the subsystem states. We also employ a traditional artificial neural network (ANN) to capture the chemical structural features of drugs. DrugGene generates final drug response predictions by combining VNN and ANN and integrating their outputs into a fully connected layer. The experimental results using drug sensitivity data extracted from the Cancer Drug Sensitivity Genome Database and the Cancer Treatment Response Portal v2 reveal that the proposed model is better than existing prediction methods. Therefore, our model achieves higher accuracy, learns the reaction mechanisms between anticancer drugs and cell lines from various features, and interprets the model’s predicted results.

**Conclusions:**

Our method utilizes biological pathways to construct neural networks, which can use genotypes to monitor changes in the state of network subsystems, thereby interpreting the prediction results in the model and achieving satisfactory prediction accuracy. This will help explore new directions in cancer treatment. More available code resources can be downloaded for free from GitHub (https://github.com/pangweixiong/DrugGene).

**Supplementary Information:**

The online version contains supplementary material available at 10.1186/s12859-024-05669-x.

## Background

Owing to the widespread application of machine learning and deep learning, biomedical science has also overcome several challenges with the help of artificial intelligence, such as cancer treatment and drug sensitivity prediction. Prediction of cancer treatment response is an important topic in clinical and pharmacological research, as people expect it to customize effective treatment plans for individual patients. However, due to the heterogeneity of tumors, patients with the same tumor type may have different treatment responses. Therefore, selecting effective drugs for patients is significant in cancer research. Since most deep learning models are black-box and lack a deep understanding of the rules behind the underlying network, drug therapy becomes more difficult [1]. Therefore, enhancing the interpretability of the model and understanding the molecular pathways that control or reflect drug sensitivity can help determine which cancer patients should receive treatment and which specific drugs have actual positive catalytic effects.

Mainstream biomedical image disease diagnosis and electronic medical record interpretation are essential in drug treatment plans in biomedicine, with machine learning models also playing an auxiliary role in predicting drug reactions [2]. Researchers have already used genomic characteristics of cell lines or tissue samples as input to predict the cellular activity of drug responses. For example, Guo et al. utilized regularization techniques based on Lasso regression to effectively control the model’s interpretability, improving its overall predictive performance [3]. Iorio et al. [4] established an elastic network model to predict cancer cell line drug IC50 (a widely used classic indicator) based on gene mutation and expression levels to observe reasonable prediction levels. Note that the elastic network absorbs the advantages of Lasso regression. Thus, the model obtained by training the elastic network can be as sparse as Lasso regression and has excellent regularization ability. Corte et al. [5] proposed the random forest model, which is associated with the statistical confidence level and improves the prediction performance based on elastic network prediction. Deep learning has achieved further success based on machine learning, with deep neural networks (DNN) [6] and variational autoencoders (VAE) [7] already applied to predict drug treatment responses demonstrating significant performance improvements for different drugs and disease conditions.

Although black-box models are undoubtedly useful, they are insufficient when it is necessary to simulate system functionality and structure. In particular, many applications in biology and medicine attempt to model functional outcomes and their production mechanisms to understand and manipulate these outcomes through drugs, genes, or the environment. However, black box models cannot be directly observed, and thus it is challenging to explain the relationship between network models and cellular molecular feature functions without understanding or paying attention to the biological mechanisms behind the predicted results. In order to overcome such limitations, model interpretability has become a research focus and a rapidly growing subfield in machine learning, with many models that achieve high prediction and description accuracy have begun to emerge [8]**.** Researchers have tried to use deep learning models in terms of interpretability. One of the main strategies is to add a modular structure to the model using prior knowledge or data and then interpret it. This strategy mapped thousands of measured cell molecular characteristics into functional modules to indicate the module's status through changes in the cell's gene expression level [5, 9]. For instance, a recent study mapped primitive molecular characteristics to a defined set of metabolic pathways. The status of these pathways predicts antibiotic resistance in cellular tissues, and specific pathway characteristics become candidate mechanisms for drug resistance [9, 10]. There are also other studies illustrating this approach, which analyzes a large set of leukemia expression profiles to extract these expression data as a set of functional gene modules, and these modules are used as interpretable features for drug response prediction [11]. In addition to model-based approaches, the other major strategy for improving the interpretability of models is an in-depth analysis of model features or feature weights to explain potential drug response mechanisms [4, 8, 12]. For example, in a black box neural network model, each input gene is assigned a weight for gene set enrichment analysis [13, 14], and then pathways that can regulate and predict drug reactions are identified [15]. However, these two strategies are not used in the modeling process, so there is insufficient experimental evidence to verify that drug response pathways can explain the internal drug response mechanisms.

In order to achieve transparency and visibility in deep neural networks, researchers have proposed some interpretable models, such as Ontotype, a universal system based on ontology for genotype to phenotype translation [16]. This study outlines the general strategies for developing computational unit models and demonstrates that ontology structures can be used functionally to explain genetic variations in phenotype prediction. However, the potential risk of this model is that predicting in a dataset may lead to overfitting, which should be effectively avoided. Ma et al. have proposed DCell, it can capture almost all phenotypic variations in cell growth and simulate the intermediate functional states of thousands of cellular subsystems. It can be further enhance the predictive results of drug sensitivity by drawing on the state analysis of this subsystem and improving the linear regression method of functional modules [17]. Recently, to perceive the inherent relationship between the network structure and biological function of deep learning models, researchers have developed DrugCell to address the lack of interpretability, which is an interpretable human cancer cell deep learning model [18]. Nevertheless, this model only inputs gene mutation data from cell lines. It should be noted that copy number variation and gene expression data have advantages compared to gene mutation data. First, gene copy number variation affects gene expression, phenotypic difference, and phenotypic adaptation by changing Gene dosage and Regulator gene activity, thus leading to tumorigenesis. Copy number variation detection can detect large DNA sequence variations in the genome as early as possible, providing a basis for diagnosing and treating diseases. This is because copy number variation is associated with the pathogenesis or susceptibility of many complex genetic diseases. Second, in most models involved in the DREAM challenge, gene expression microarrays provide greater predictive power than other data types. In approximately 90% of the models, gene expression is used alone or in combination with other feature types, including mutations, CNV, methylation, and RPPA. Therefore, we also consider adding data for these two genotypes.

This study develops DrugGene, a model that combines visible neural network (VNN) [17, 19, 20] embedded in the hierarchical structure of biological processes with traditional artificial neural network (ANN) [17, 21, 22], which simulates the reaction process of human cancer cells to drug therapy. VNN receives gene mutations, gene expression, and gene copy number variation data from cell lines, generating an output in the neural network's final layer. At the same time, the fingerprint-encoded data of the drug is inputted into ANN and outputted through a multi-layer network. Then the outputs of the two branches are integrated to obtain the predicted target value. The predicted values can be used to analyze the final drug reaction results. DrugGene predicts the response of genotype level changes to cell growth by mapping VNN neurons to pathways established by hierarchical structures while identifying highly correlated molecular pathways that drive these predictions, achieving mechanical and transparent interpretability. Finally, the experiment proves that the proposed model improves the predictive performance of drug sensitivity compared to DrugCell on the same test set.

## Methods

### Dataset filtering and preprocessing

In order to verify the proposed model, we screened four cancer resource databases: Cancer Treatment Response Portal (CTRP) [23] (http://portals.broadinstitute.org/ctrp), Cancer Cell Line Encyclopedia (CCLE) [24–26] (https://depmap.org/portal/download/all), Cancer Drug Sensitivity Genome (GDSC) [18, 27] (https://www.cancerrxgene.org) and Gene Ontology (GO) [28] (https://www.informatics.jax.org/vocab/gene_ontology) databases. We used CTRP, which links the genetic, lineage, and other cellular characteristics of cancer cell lines with small molecule sensitivity, to accelerate the discovery of treatment methods that match cancer patients. The experiment included 684 drugs, 942 cell lines, and 8969 cell line-drug pairs. The target value is the area under the dose–response curve (AUC). We retrieved and screened compound data from GDSC and CTRP to obtain SMILES notation based on the drug names provided in the dataset. On the other hand, we extracted genomic data required for cancer cell lines from CCLE and GDSC as characteristic data for the cell line. Genomic data include gene mutation data, gene expression level data, and gene copy number data. The Gene Ontology (GO) database contains information on molecular function, cellular components, and biological processes, from which 2086 biological process information was selected for our model branch modeling. The detailed information about experimental datasets can be found in Table 1.

**Table 1:** Experimental datasets on cell lines, drugs, and gene ontology

	Name	Quantity	Data form	Database
Cell lines	Cell line	684	gene mutation, gene expression, and gene copy number variation	CCLE GDSC
Drugs	SMILES	942	Morgan fingerprint	GDSC, CTRP
Cell line-drug pairs	AUC	8969	AUC	CTRP
Gene ontology	Biological process	2086	GO Term	GO

**Fig. 1:**
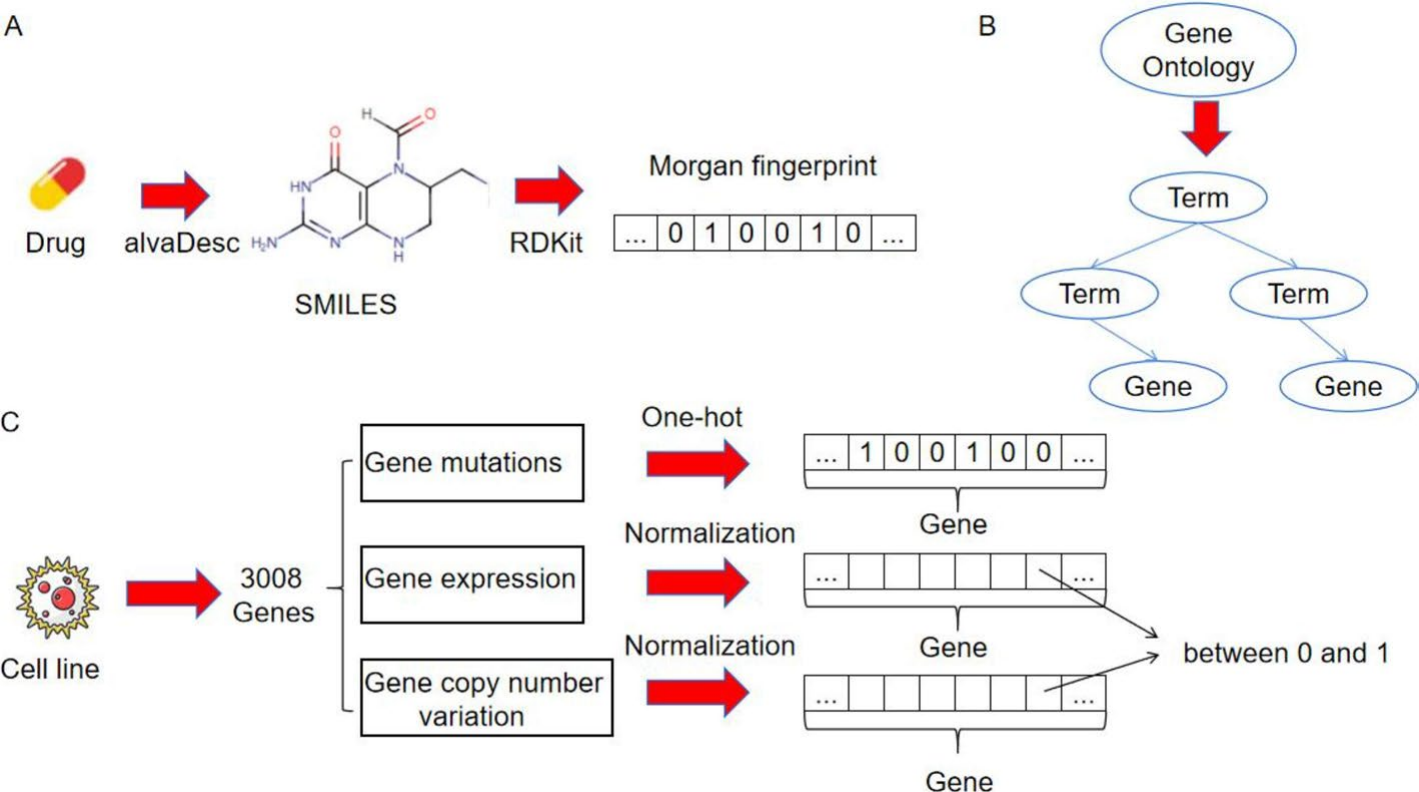
**A** The processing process of drug data. Obtain the SMILES symbol and Morgan fingerprint code with a length of 2048 for each drug, **B** Select available biological process information from Gene Ontology (GO), **C** The preprocessing process of cell line data, from which available gene mutation, gene expression, and gene copy number variation data can be obtained

In order to ensure that the data format conforms to the specifications of deep learning models, the data preprocessing is carried out here. For each drug, to facilitate data input into the neural network, we used the software alvaDesc (https://chm.kode-solutions.net/products_alvadesc.php) to calculate the molecular descriptors of compounds, and we used RDKit (http://www.rdkit.org) to calculate the Morgan fingerprint encoding of the drug, which iteratively obtains different pathways for each atom in the molecule and decomposes the chemical structure of the drug into molecular fragments. Each fragment is represented as a 2048 vector containing 0 and 1 (Fig. 1A). For genomic data of cell lines, and we selected the top 15% of genes most commonly mutated in human cancer based on CCLE and the genes annotated in the GO database. Thus, 3008 genes were screened, where each cell line corresponded to a vector of 3008 and each element to a gene (Fig. 1C). Due to the composition of gene mutations, gene expression, and gene copy number variation datasets extracted from public datasets, there are situations where one or more data features are missing. Therefore, we supplemented or performed special processing before conducting feature fusion for gene data that was not included. Our approach used the average genotype data corresponding to the cell line to replace missing gene data. The gene mutation data of cancer cell lines was in text format, encoded using one-hot encoding, where 1 represented the gene mutation, and 0 represented no mutation. Similarly, the gene expression level and gene copy number variation data of cancer cell lines were also in text format, and numerical encoding was performed using a maximum and minimum range normalization method. To be specific, the values are scaled to the range of 0–1 and then used as the characteristic values of the cell line. Finally, each data item in the screening data set is compared to form a characteristic tensor of medicinal chemistry characteristics and cancer cell lines. We differentiated between gene-tagged terms and non-gene-tagged terms from the Gene Ontology (GO) datasets and established a hierarchical connection between parent and child nodes based on the inclusion relationship in the biological ontology (Fig. 1B). The preprocessing results of the experimental dataset are shown in Additional file 1: Table S1.

### Model architecture

**Fig. 2:**
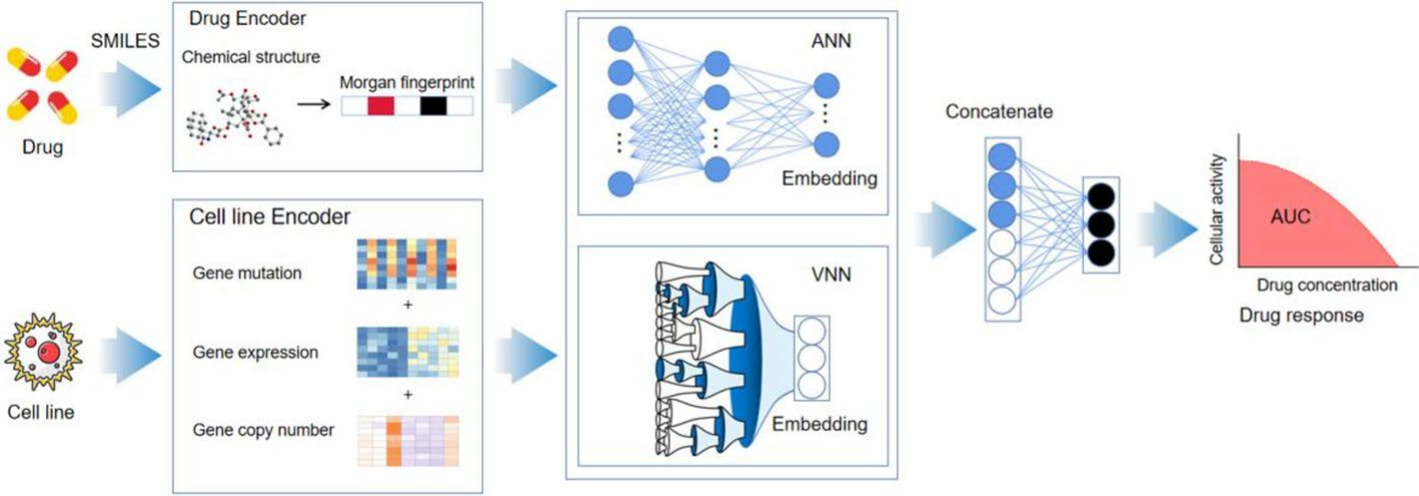
Workflow of DrugGene. DrugGene uses visible neural networks (VNN) and traditional artificial neural networks (ANN) as sub-modules and combines their outputs for drug response prediction

Regarding the design of DrugGene, the model was designed as a neural network with two branches. One involved a visual neural network (VNN) that modeled the hierarchical organizational structure of molecular subsystems in human cancer cells, VNN receives inputs of gene mutation, gene expression, and gene copy number variation data from cell lines, all of which are matrices with the same dimensionality. We can fuse them into a new matrix through the superposition operation of tensors for input into VNN without changing the dimensions. The other branch was a traditional artificial neural network (ANN) that received the Morgan fingerprint encoding input for drugs. The VNN and ANN sub-models are trained independently during the training phase. Through the collaborative work of the branches, the two branch networks were connected to combine their outputs into a layer of neurons and finally integrated to produce a predicted drug sensitivity response result (Fig. 2).

### Description of VNN in the model

VNN models the hierarchical structure of human cell molecular subsystems based on the biological process information recorded in the Gene Ontology database. We extracted 2086 biological processes from them to construct the cellular subsystem of VNN.

**Fig. 3:**
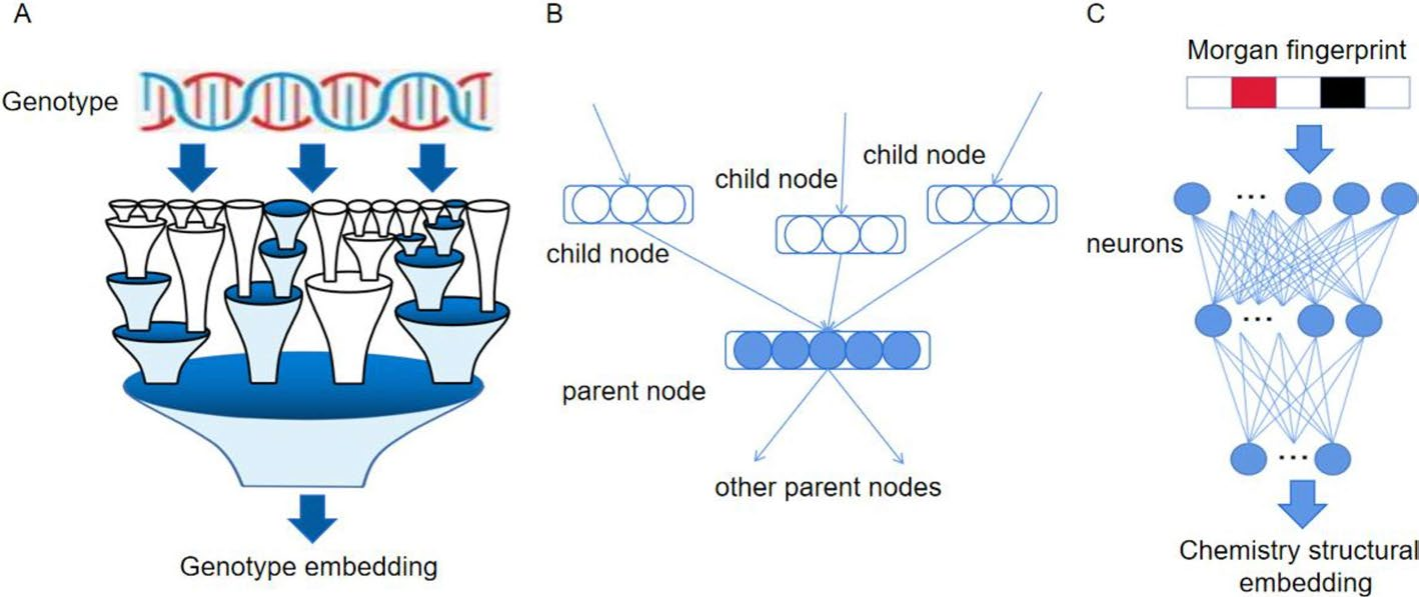
**A** In visible neural networks, genotype inputs are transformed into genotype embedding through the hierarchical structure of cellular subsystems, **B** The subsystem nodes in a visual neural network are assigned a set of neurons, which are interconnected through parent–child node relationships, **C** Artificial neural networks receive input from fingerprint encoding of drug chemical structures and generate embedded representations of chemical structures

The subsystems in these ontologies exist as nodes in neural networks and are interconnected through common node relationships or hierarchical parent–child node relationships. Each subsystem is assigned a set of neurons to represent its functional state, making the cellular subsystem multifunctional (Fig. 3B). Their connectivity is reflected in the hierarchical structure of organisms, e.g., from small complex reactions to larger signal pathways, ultimately reaching the overall function of the cell. Therefore, neurons only receive input information from the neurons of the child node and only send output to the neurons of the parent node. The network weight is determined during the training process. A network structure consisting of 2086 subsystems was designed by layering the subsystems, with a maximum connection depth of six subsystems, to define VNN branches embedded in genotype information (Fig. 3A). This branch structure constructs a robust bridge from changes in genotype status to changes in cell activity or drug sensitivity.

The genomic data of human cell lines, including gene mutations, gene expression, and gene copy number variation, were used as the input of the VNN. The genomic data of these three types are represented by two-dimensional tensors of length 3008. We use the superposition operation of tensors to merge them into a new tensor, while normalizing the data and scaling the tensor values to between 0 and 1. Then VNN will generate a placeholder node to store and read this input tensor. The Gray code conversion is a reliable encoding method that minimizes errors [29, 30]. It effectively prevents the phenomenon of using binary encoding that may deviate from the optimal solution and fail to achieve stability. Therefore, we converted the binary encoding of gene mutations into Gray codes. The output layer obtained the calculated genotype embedding data to represent the embedding state of the entire cell based on the genotype. In addition, the 3008 genes were organized into a hierarchical structure of nested gene sets based on terms extracted from the hierarchical structure of biological processes to represent different functional cellular subsystems [17]. Given that gene perturbations propagate through the hierarchical structure of the subsystems they are contained, it may lead to functional changes in cellular subsystems, generating predictive responses to cell activity levels. In order to achieve transparent and visible biological explanations, we directly embed the structure of deep neural networks into the biological hierarchy. VNN can use the genotype of cell lines to monitor changes in the status of network subsystems and reflect the importance of pathways, thereby interpreting the prediction results in the model. By enhancing the analytical ability of VNN, the model performance can be better improved.

To train the model, the training process is performed by minimizing the objective function, randomly initializing all weights between -0.01 and 0.01, and using the Batch Normalization function to reduce the impact of internal covariate shifts caused by different weight scales. We set the training dataset to $D=\left\{\left({X}_{1}, {Y}_{1}\right),\dots ,\left({X}_{N}, {Y}_{N}\right)\right\}$, where $N$ is the number of samples, for each sample $i$,${X}_{i}\in {R}^{M}$ represents genotype through a binary vector of states on $M$ genes, and ${Y}_{i}\in R$ is a numerical value representing the observed drug response. The multidimensional state of each subsystem $t$ is represented by the output vector ${O}_{i}(t)$, denoted by a linear function of all its subsystems and annotated gene states, connected to the input vector ${V}_{i}(t)$:

1 $${O}_{i}\left(t\right)=Batch Normalization (Tanh(W\left(t\right){V}_{i}\left(t\right)+b(t)))$$

In Formula (1), $Batch Normalization$ is a regularization of model weights, which can solve gradient vanishing and reduces traditional drop out steps in deep learning [17] and $Tanh$ is a nonlinear transformation hyperbolic tangent function. We perform the training process by minimizing the objective function:

2 $${\frac{1}{N}\Sigma }_{i=1}^{N}Loss\left(linear({O}_{i}\left(r\right), {Y}_{i})\right)+\alpha {\Sigma }_{t\ne r}Loss\left(linear({O}_{i}\left(t\right), {Y}_{i})\right)+\lambda {\Vert W(t)\Vert }_{2}$$

In Formula (2), $Loss$ is the squared error loss function, and ${\text{r}}$ is the root of the hierarchy. ${{\text{O}}}_{{\text{i}}}\left({\text{r}}\right)$ denotes the output of the root and ${{\text{O}}}_{{\text{i}}}\left({\text{t}}\right)$ represents the output of other subsystems. By linear transformation function, each subsystem is optimized to feature its parent node and predict its action value. $\uplambda$ is the regularization factor of L2 norm determined by four-fold cross validation. In addition, selecting appropriate learning rate parameters $\mathrm{\alpha }$ can make the objective function converge to a local minimum in an appropriate time. For model optimization, ADAM is a popular random gradient descent algorithm commonly used to optimize the objective function during the training process [31–33], with a minimum batch size of 10,000. The learning rate is determined in the range of ${10}^{-1}$, ${10}^{-2}$, ${10}^{-3}$ and ${10}^{-4}$ through grid search. The gradients related to model parameters are calculated using standard backpropagation.

### Description of ANN in the model

The second branch system is constructed from a traditional hierarchical artificial neural network (ANN), which is inspired by the actual neural network and its processing patterns in the brain. With the extremely powerful computing power of ANN, it can accurately process high-dimensional data encoded by drug fingerprints without the extensive feature engineering [17]. Another advantage of ANN is that it can effectively avoid overfitting and achieve better prediction results. The function of ANN is created during the training phase, where model learning captures highly available information as accurately as possible and returns the correct output answers for each sample input. We use $X$ to denote features or known conditions, and $Y$ to denote labels or results. Here is the basic formula:

3 $$Y=WX+b$$

The training of artificial neural networks is actually achieved by continuously modifying the weight vector $W$ and bias $b$ through algorithms to approximate the real model as much as possible, in order to achieve the best prediction performance of the entire neural network. A loss function has been defined:

4 $$Loss={(p\left\{Y\right\}-t\{Y\})}^{2}$$

In formula (4), $p\left\{Y\right\}$ is a numerical value representing the predicted value of the sample, and $t\{Y\}$ is a numerical value representing the true value. The goal is to make the predicted value $p\left\{Y\right\}$ as close as possible to the true value $t\{Y\}$. The loss function is to minimize the sum of the loss values of a neural network as much as possible. The training should be terminated and the parameters of the trained neural network saved when the loss function reaches a certain convergence threshold.

We embedded the Morgan molecular fingerprint code of the chemical structure of drugs in the ANN branch system, which is a typical vector representation of the chemical structure [34]. Drugs are represented by SMILES symbols. The Morgan fingerprint code of each drug is represented by a binary vector with a length of 2048. Each element in the fingerprint represents a specific activation state (0 = inactive; 1 = activated), and the fingerprint carrier is represented by an average of 81 activation sites, with each site typically representing less than 10 molecular fragments. In the model, the ANN is set to three layers, each layer can be assigned a certain number of neurons (Fig. 3C). Morgan fingerprint encoding serves as the input for the first hidden layer of ANN, and the data information propagates between layers in the network. The last layer of the network generates an embedded representation of the drug chemical structure, which is also the prediction result of ANN.

### Full connection between VNN and ANN

The fully connected layer is responsible for converting the computed features of the network into a tensor, and its advantage is to reduce the influence of feature positions on the regression results. The genotypic embedding generated by VNN and medicinal chemistry structural embedding generated by ANN are fully connected to establish a complete model network structure, and the predicted response value is calculated to obtain the predicted response result of drug sensitivity. In general, the full connection form is employed by concatenating the output tensors of VNN and ANN along a specified dimension to generate a novel tensor. The utilization of this methodology is widely prevalent in the field of deep learning [18]. The area under the normalized dose response curve (AUC) is used as the target value. AUC = 0 indicates complete cell killing, and AUC = 1 indicates no effect.

## Results

### Performance evaluation of DrugGene in predicting drug sensitivity

**Fig. 4:**
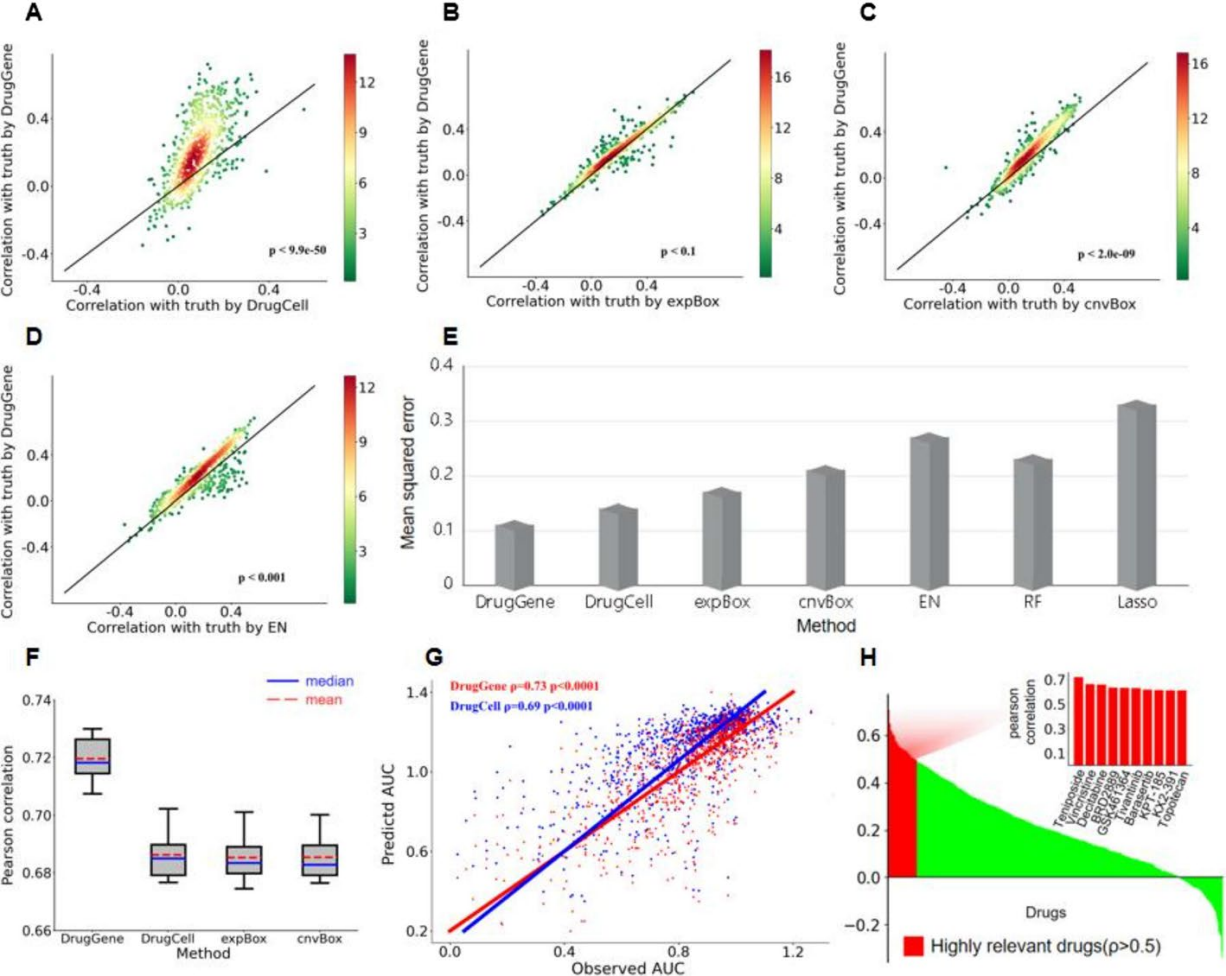
**A–D** Comparison of drug sensitivity prediction performance between DrugGene and four existing models: **A** DrugCell, **B** expBox, **C** cnvBox, and **D** elastic net. The number of drugs participating in the reaction is 684, and the number of cell lines is variable. The points represent each drug, and the points above the diagonal show that DrugGene has a higher prediction accuracy than other models. **E** MSE comparison subgraph for prediction methods, **F** Box plot of drug reactions established in all experimental data demonstrating the predicted drug sensitivity intervals between DrugGene and three types of DrugCell models, **G** Scatter plot of predicted values for DrugGene and DrugCell, and **H** Waterfall chart revealing the ranking of the predictive performance of each drug. The red part distinguishes drugs with higher prediction accuracy (*p* > 0.5). The mapped bar chart shows the top 10 drugs with predicted correlation rankings

This study used a tenfold cross-validation method to evaluate the predictive accuracy of DrugGene based on the average Pearson correlation coefficient between the predicted and the observed AUC values in the experimental data, which were employed as the prediction result. In order to verify the predictive performance of the proposed method, DrugGene was compared with current models on the same dataset. Specifically, during the training phase, 684 drugs, 942 cell lines, and 8969 cell line-drug pairs were used to train models. In addition, during the testing phase, we still screened the reaction results of same cell line-drug pairs to test and evaluate these models separately. Compared to DrugCell, DrugGene has a significantly higher prediction accuracy for predicting the response of a single drug because DrugCell utilizes gene mutations and drug characteristics for drug sensitivity prediction. On this basis, DrugGene can effectively improve the prediction results by integrating gene mutation, gene expression, gene copy number variation, and Medicinal chemistry characteristics (Fig. 4A).

Moreover, we conducted two comparative experiments simultaneously, and specifically, we combined gene expression or gene copy number variation data with medicinal chemistry characteristics and then used that data to train two comparison models. The model which only uses gene expression and medicinal chemistry features as input are called expBox and the other model that only uses copy number variation and drug coding data as input are referred as cnvBox. The experimental results indicated that DrugGene has better predictive performance than these two models (Fig. 4B, C). Among them, the difference between DrugGene and expBox is smaller compared to cnvBox and DrugCell. For the elastic network (EN), this method is also often used to predict drug sensitivity because of its advanced regression technology. Elastic network is a linear regression models trained using L1 and L2 norms as prior regularization terms [35, 36]. Additionally, we compared the prediction results of DrugGene and elastic network, demonstrating that DrugGene's prediction accuracy is higher than in over 80% of drug reactions (Fig. 4D).

We created a box plot to compare the predictive performance of these four methods (Fig. 4F), and a tenfold cross-validation method was used to evaluate the Pearson correlations of these models. Figure 4F highlights DrugGene's predictive correlation is significantly higher than the competitor models, which have relatively close median values. Hence, feature fusion can achieve higher prediction accuracy than a single feature. The significant difference levels between DrugGene and DrugCell, expBox, and cnvBox are 2.8e−08, 1.1e−08, and 1.2e−08, respectively.

We compared the prediction performance of seven models (Fig. 4E) and measured their regression performance using the Mean squared error (MSE) metric. MSE is the square of the difference between the predicted value and the true value, which is a commonly used performance indicator in regression problems [37]. Figure 4E shows the MSE comparison results of seven models on the test set. The MSE of regression performance indicators of seven models, DrugGene, DrugCell, expBox, cnvBox, elastic network, Random forest (RF) [38] and Lasso regression [39, 40], were 0.11, 0.14, 0.17, 0.21, 0.27, 0.23 and 0.33 respectively. The results infer that our method has the best predictive performance, followed by DrugCell, with a reduction rate of 21.4% compared to it. In addition to using Pearson correlation coefficient and MSE, we also consider using more regression evaluation indicators, including coefficient of determination (R^2^), mean absolute error (MAE), and root mean square error (RMSE), to comprehensively evaluate the predictive performance of the model. As shown in Additional file 1: Table S2, the table displays the comparison results of all models based on the use of five regression evaluation indicators. It reveals that DrugGene's predictive performance on Pearson correlation coefficient, MSE, MAE, and RMSE is superior to other models, second only to expBox in R^2^. Therefore, the proposed DrugGene model demonstrates an improved regression performance. Indeed, we plotted a visual scatter using the predicted values of DrugGene and DrugCell on the test set, revealing that DrugGene has a better fitting performance than DrugCell (Fig. 4G).

Finally, the waterfall chart illustrates the Pearson correlation of the predicted reactions of 684 drugs, with the predictive performance arranged from high to low, the horizontal axis representing the drugs, and the vertical axis representing the evaluation indicators (Fig. 4H). The red illustrations show the top ten drugs with the highest prediction accuracy, where the drug with the highest score is teniposide. Teniposide is a chemotherapy drug mainly used to treat acute lymphocytic leukemia in children. The second highest scoring is vincristine, which has good anti-tumor effects. Currently, its formulation is used as a clinical anti-tumor drug and is often used for the treatment of acute leukemia, especially in children. For compounds that can be highly predicted by DrugGene, they often target different targeted therapies and can trigger a larger range of cellular reactions, indicating that the predicted results of the model can reflect the therapeutic effects of specific targeted drugs.

### Learning the mechanisms of drug reactions through DrugGene

**Fig. 5:**
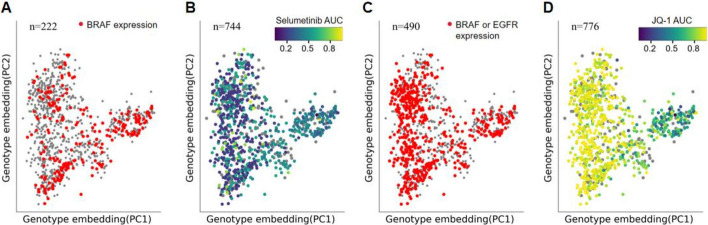
**A-B** Genotype embedding of the cell line, with the x-axis and y-axis representing the first two main components selected. **C-D** Similar to Figures **A** and **B**, the color in Figure **C** distinguishes the expression level of BRAF or EGFR, while the color in Figure **D** distinguishes the sensitivity and resistance of drug reactions

After evaluating DrugGene's predictive ability based on the treatment response of each drug, we discuss the model’s interpretability. The transparency and mechanical interpretability were aided by VNN and ANN in the model, respectively. For cell line's drug response, we analyzed the impact of cells on drug sensitivity or resistance based on the expression levels of specific genes within the cells. Here, the two-dimensional visualization results of each cell line can be intuitively observed by extracting the two main components from all genotype data generated by VNN. The points in Fig. 5A are drawn based on the extracted principal components; each point is a cell line. Figure 5A reveals the distribution of specific genotype expression levels that lead to different drug sensitivities, such as coloring based on the expression level of BRAF, points with higher expression levels are set to red, otherwise set to gray. The results reveal that BRAF with high expression levels promotes sensitivity to the MEK inhibitor seluminib (Fig. 5B). This result is visualized on a two-dimensional plane where the AUC value of the drug reaction is color-coded. A smaller AUC value indicates sensitivity, while a higher one indicates resistance. Combining Fig. 5A and B reveals that most of the sensitive cell lines in Fig. 5B correspond to the red dots in Fig. 5A. Besides, we find that Seluminib is an inhibitor for BRAF mutations in clinical treatment [41]. We also analyze the interpretability of the model when the cell lines exhibit drug resistance. Figure 5C distinguishes the distribution of EGFR or BRAF expression levels. Similarly, the points with higher expression levels are highlighted in red. For EGFR or BRAF, high expression levels can confer resistance to the BET family inhibitor JQ1 (Fig. 5D). The points presented as drug resistance mostly correspond to the red points in Fig. 5C. In clinical treatment, JQ1 is often used as an inhibitor for EGFR or BRAF mutations.

**Fig. 6:**
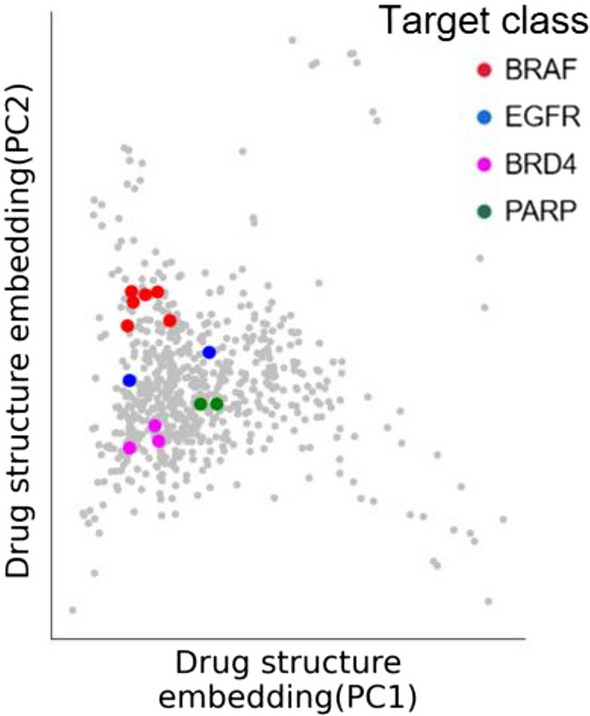
Drug structure embedding. Points are drugs, and colors represent specific targeted drugs

Regarding the medicinal chemistry structure embedding obtained from ANN, we selected two main components to visualize the targeted drugs two-dimensionally, where each point represents the drug (Fig. 6). The results indicate that in the drug targeting category, drugs can be layered according to the different mechanisms of action of targeted genes, and the drugs with different genes as targeted genes in the figure exhibit a clustering phenomenon. For example, the target genes that exhibit good clustering performance in the graph include BRAF, BRD4, and PARP, which are labeled with different colors. In clinical trials, these targeted drugs have been proven to act as inhibitors targeting these targeted genes [42]. In summary, DrugGene is able to distinguish key features of genotypes that lead to drug sensitivity and resistance, as well as understand the chemical structural characteristics of drug biological activity.

### The role of subsystems in neural networks

Due to the hierarchical structure of the subsystems extracted from biological processes in human cells, VNN's genotypic output can reflect the state changes of specific subsystems in the network structure. Then we can distinguish important subsystems with prominent predictive functions through these state changes. Specifically, we used the relative local improvement in predictive power metric (RLIPP), which evaluates the performance of these subsystems based on the degree of predicted drug response of the parent node relative to the child node in VNN. Thus RLIPP determines the subsystem with the best predictive performance [17, 18]. Additionally, we used neuron values representing the states of the parent and child nodes to predict drug response, respectively. The performance evaluation indicator is the Pearson correlation coefficient between the predicted values of the parent or child nodes and the actual target values. Then RLIPP was defined as the degree to which the predicted value of the parent node improves relative to the predicted value of the child node:

5 $$RLIPP=\frac{{P}_{2}-{P}_{1}}{{P}_{1}}$$

where ${P}_{1}$ represents the Pearson correlation coefficient predicted by the child node, and ${P}_{2}$ represents the predicted result of the parent node. Specifically, for parent nodes connected to multiple child nodes, we compare the predicted results of the parent node with the average of the predicted results of these child nodes. $RLIPP>0$ indicates that the parent node has a more prominent predictive ability than the child node, while conversely, the child node has a stronger predictive ability. Therefore the RLIPP score indicates the importance of the parent–child system during prediction.

**Fig. 7:**
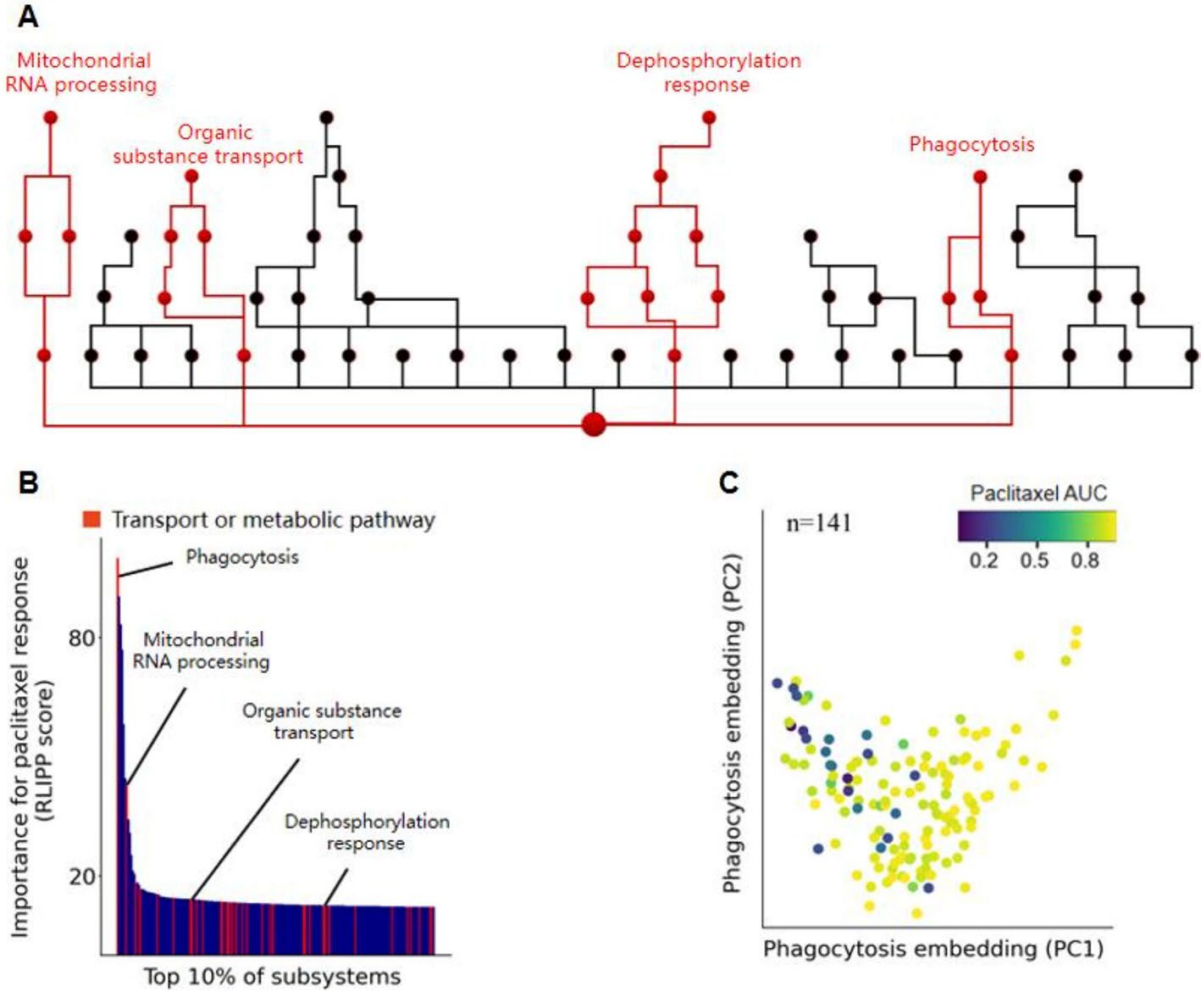
**A** Subsystem network structure, with red branches representing transport and metabolic pathways. **B** Waterfall plot of the top 10% subsystems with significant RLIPP scores for paclitaxel response. The subsystems representing transport and metabolic pathways are highlighted in red. **C** The phagocytosis subsystem with the highest RLIPP score responds to paclitaxel. The point is the cell line. The smaller the AUC value, the more sensitive the cell response appears, and vice versa, the more resistant it appears

We chose paclitaxel to react with cells and used the RLIPP score to evaluate the important subsystems in this reaction process (Fig. 7B). Figure 7B illustrates the subsystems with the highest scores in the top 10% (including 200 subsystems). The red section indicates transport or metabolic pathways, and most of the first 200 subsystems belong to these two categories of pathways. The results indicate that these pathways have relatively outstanding predictive abilities. In order to observe these pathways and the overall network architecture more clearly, Fig. 7A provided the two-dimensional visualization of a portion of VNN, and the important pathways are highlighted in red, including the Phagocytosis with the highest score and other pathways in Fig. 7B, such as Mitochondrial RNA processing, Organic substrance transport, and Dephysiological response.

In the reaction process of paclitaxel, we used the state changes of the subsystems with the highest scores to represent the predicted values of drug reactions and found that the higher-ranked Phagocytosis subsystem could distinguish the sensitivity and resistance of cell lines reacting with paclitaxel (Fig. 7C). The lower the AUC value, the more sensitive the response, while the opposite indicates the drug resistance response.

## Discussion

Machine learning and deep learning technologies effectively predict drug response, but due to the lack of interpret the predicted results of these methods, it is difficult to effectively explain the internal working mechanism of the model used and the role of its network. The proposed DrugGene combines the biological processes of human cells, artificial neural networks, and the working mechanisms within the model to form a complete network structure. DrugGene can receive encoded data from cell lines and drugs to predict the drug response in cancer cells and effectively design targeted drug therapy methods. Especially, DrugGene is able to read more abundant genotype data as data input. Compared to DrugCell which only uses gene expression data, our model has added gene expression and copy number variation data. Gene expression data contains information about gene activity, which can reflect the current physiological state of cells. It is helpful to elucidate the gene expression regulatory pathways and regulatory networks. Moreover, by calculating the copy number of genes, significant DNA sequence variations in the genome can be effectively discovered, thereby further understanding the relationship between genes and diseases and providing important basis for disease diagnosis and treatment. In terms of network construction, the VNN in DrugGene has a greater neural network depth compared with DrugCell. For neural networks, the more hidden layers in neural networks and their derived types of networks, the higher the level of abstraction of input features. This means that neural networks can learn more feature representations of data on certain specific tasks, thus more accurately describing the essence of input data. During the model training phase, DrugGene uses the Batch Normalization and gradient accumulation functions to improve the speed of model training compared with DrugCell. This not only effectively avoids vanishing and exploding gradients, but also allows for higher learning rates. Meanwhile, we further optimized our model. The ADAM optimizer can dynamically update parameters and be used to optimize the objective function. It is combined with stochastic gradient descent (SGD) to optimize function convergence. The experimental results indicate that our method has indeed improved predictive performance. Therefore, DrugGene's VNN can effectively reflect the interpretability of the model's prediction results and the biological mechanisms behind drug reactions, providing a solution for constructing interpretable biomedical prediction models.

DrugGene can monitor changes in the network’s subsystems state using the genotype of the cell line, thereby explaining the predicted results in the model. Due to combining these biological pathways and deep neural networks, DrugGene achieves better prediction results. In future research, researchers can reasonably use the continuously updated deep learning model and more prominent neural network and combine the biological pathway, the corresponding genotype characteristics, and medicinal chemistry characteristics to achieve better performance on drug sensitivity prediction. Different feature preprocessing strategies may be used to improve the model's predictive performance, such as encoding optimization and feature fusion methods. The model's predictive performance may be further improved through these operational methods. In addition, using heterogeneous bioinformatics network models can learn latent information from interaction networks and make more accurate predictions [43, 44]. This type of algorithm can remove noise from biological data, extract functional information of drugs and genes, and improve prediction accuracy through feature learning algorithms.

## Conclusion

The method DrugGene proposed in this article is based on partial reference information, and its effectiveness is more in line with practical clinical practice. It not only effectively integrates reference data from cell lines and drugs, but also constructs a portion of the network using gene ontology data, making the model interpretable and achieving satisfactory drug sensitivity prediction accuracy, which is beneficial for reducing medical costs, analyzing new strategies for cancer drug treatment, and providing certain assistance for cancer immunotherapy.

### Supplementary Information

**Additional file 1**. **Supplementary Table 1.** Preprocessing results of experimental dataset.

## Data Availability

The relevant code resources and datasets can be downloaded from GitHub (https://github.com/pangweixiong/DrugGene).

## Abbreviations

VNN: Visual neural network
ANN: Artificial neural network
GO: Gene Ontology
CCLE: Cancer Cell Line Encyclopedia
GDSC: Cancer Drug Sensitivity Genome
CTRP: Cancer Treatment Response Portal
MSE: Mean squared error
RLIPP: Relative local improvement in predictive power metric
