# Supplementary material for: Prediction of anticancer drug sensitivity using an interpretable model guided by deep learning
Source: BMC Bioinformatics. 2024 May 9;25:182. doi: 10.1186/s12859-024-05669-x (PMC11080240; doi:10.1186/s12859-024-05669-x)
Supplement: Supplementary file 1 — Additional file 1. Supplementary Table 1. Preprocessing results of experimental dataset. [file 12859_2024_5669_MOESM1_ESM.docx]

**Prediction of anticancer drug sensitivity using an interpretable model guided by deep learning**

**Supplementary Material**

Supplementary Table 1 Preprocessing results of experimental dataset

|  | Cell lines | Drugs | Gene ontology |
| --- | --- | --- | --- |
| Available data | gene mutations, gene expression, and gene copy number variation: binary encoding with a length of 3008 | Morgan fingerprint: binary encoding with a length of 2048 | 2086 terms with parent-child relationships |

| Supplementary Table 2 Comparison of Regression Performance of Models | | | | | |
| --- | --- | --- | --- | --- | --- |
| Method  Model | Pearson | MSE | R^2^ | MAE | RMSE |
| DrugGene | 0.72±0.01 | 0.11±0.03 | 0.44±0.04 | 0.30±0.02 | 0.33±0.04 |
| DrugCell | 0.68±0.01 | 0.14±0.04 | 0.43±0.05 | 0.34±0.04 | 0.37±0.06 |
| expBox | 0.69±0.01 | 0.17±0.02 | 0.46±0.03 | 0.39±0.05 | 0.41±0.02 |
| cnvBox | 0.67±0.02 | 0.21±0.02 | 0.40±0.01 | 0.43±0.02 | 0.46±0.02 |
| EN | 0.55±0.02 | 0.27±0.03 | 0.31±0.04 | 0.52±0.05 | 0.52±0.03 |
| RF | 0.57±0.03 | 0.23±0.05 | 0.25±0.03 | 0.56±0.04 | 0.48±0.05 |
| Lasso | 0.48±0.08 | 0.33±0.07 | 0.22±0.06 | 0.61±0.07 | 0.57±0.07 |
